# Transcranial direct current stimulation (tDCS) has beneficial effects on liver lipid accumulation and hepatic inflammatory parameters in obese rats

**DOI:** 10.1038/s41598-021-90563-2

**Published:** 2021-05-26

**Authors:** Larisse Longo, Valessa Emanoele Gabriel de Souza, Dirson João Stein, Joice Soares de Freitas, Carolina Uribe-Cruz, Iraci L. S. Torres, Mário Reis Álvares-da-Silva

**Affiliations:** 1 Graduate Program in Gastroenterology and Hepatology, Universidade Federal do Rio Grande do Sul, Porto Alegre, Brazil; 2 Experimental Laboratory of Hepatology and Gastroenterology, Center for Experimental Research, Hospital de Clínicas de Porto Alegre, Porto Alegre, Brazil; 3 Laboratory of Pain Pharmacology and Neuromodulation: Preclinical Investigations, Center for Experimental Research, Hospital de Clínicas de Porto Alegre, Porto Alegre, Brazil; 4 Graduate Program in Medicine: Medical Sciences, Universidade Federal Rio Grande do Sul, Porto Alegre, Brazil; 5 Graduate Program in Biological Sciences, Pharmacology and Therapeutics, Universidade Federal Rio Grande do Sul, Porto Alegre, Brazil; 6 Division of Gastroenterology, Hospital de Clínicas de Porto Alegre, Porto Alegre, Brazil

**Keywords:** Biotechnology, Gastroenterology

## Abstract

Obesity is key to liver steatosis development and progression. Transcranial direct current stimulation (tDCS) is a promising tool for eating disorders management but was not yet evaluated in steatosis. This study investigated tDCS’ effects on liver steatosis and inflammation in an experimental obesity model. Male Wistar rats (60 days-old) were randomly allocated (n = 10/group) as follows: standard-diet/sham tDCS (SDS), standard-diet/tDCS (SDT), hypercaloric-cafeteria-diet/sham tDCS (HDS), and hypercaloric-cafeteria-diet/tDCS (HDT). After 40 days of diet, animals received active or sham tDCS for eight days and were euthanized for liver fat deposition and inflammation analysis. HDS and HDT animals showed cumulative food consumption, total liver lipid deposits, IL-1β, TNF-α levels, IL-1β/IL-10 and TNF-α/IL-10 ratios significantly higher than the SDS and SDT groups (*p* < 0.001 for all parameters). tDCS (SDT and HDT) reduced liver lipid deposits (0.7 times for both, *p* < 0.05), IL-1β (0.7 times and 0.9 times, respectively, *p* < 0.05) and IL-1β/IL-10 index (0.6 times and 0.8 times, respectively, *p* < 0.05) in relation to sham (SDS and HDS). There was an interaction effect on the accumulation of hepatic triglycerides (*p* < 0.05). tDCS reduced 0.8 times the average liver triglyceride concentration in the HDT vs. HDS group (*p* < 0.05). In this obesity model, tDCS significantly decreased liver steatosis and hepatic inflammation. These results may justify looking into tDCS utility for human steatosis.

## Introduction

Currently, obesity reaches epidemic proportions, being widely disseminated, particularly in urban settings, and has clinical implications with potential negative effects on almost every organ system, representing a psychosocial and economic burden^1^. The management of obesity is often difficult or unsuccessful because it has a multifactorial nature. There is significant evidence that high body mass index is a risk factor and is associated with the development of a growing set of chronic diseases, including metabolic associated fatty liver disease (MAFLD), cardiovascular disease, insulin resistance, diabetes and chronic kidney disease^2–4^. In this regard, randomized clinical trials demonstrate that weight loss interventions (diet and exercise) are able to reduce the serious damage to health and mortality rates in obese adults^5^.

Chronic low-grade inflammation caused by obesity has been reported in several organs^6^. In the liver, the accumulation of lipids in hepatocytes can induce a subacute inflammatory response similar to that observed with the accumulation of lipids in adipocytes^7,8^. MAFLD, a new terminology for non-alcoholic fatty liver disease (NAFLD) is a spectrum of liver diseases that encompasses simple fatty infiltration in > 5% of hepatocytes (steatosis) in patients with metabolic abnormalities, regardless of the amount of alcohol consumption or the presence of comorbidities, like chronic viral hepatitis^9,10^. This spectrum may evolve to non-alcoholic steatohepatitis, which is characterized by hepatocyte ballooning, lobular inflammation and fibrosis, that could worsen into cirrhosis and hepatocellular carcinoma^7^. MAFLD affects over 20% of people in the West, and up to one third of people living in South America or the Middle East^7^. Despite the growing impact of this clinical condition on public health, treatment options remain limited and there are no therapies approved by the Food and Drug Administration (FDA)^11,12^.

Transcranial direct current stimulation (tDCS) is a non-invasive technique that modulates cortical excitability in the human cortex^13^. This technique consists of applying a weak, constant, low intensity electric current between two electrodes over the scalp in order to modulate cortical excitability^13^. While anodal stimulation of the motor cortex increases neuronal activity, cathodal stimulation inhibits it^13^. The mechanisms by which tDCS induces changes across different levels of the nervous system may involve membrane polarization and, consequently, the modulation of neuronal activity^14^. In this context, this technique has been used in different clinical contexts, with electrodes applied to specific areas of the brain according to the expected result^15–18^. Studies indicate that tDCS can suppress food craving in rodents and humans and this effect may be involved with the cortical areas related to decision-making, having the dorsolateral prefrontal cortex (DLPFC) responsible for mediating the mechanisms of food reward^14,19–22^. Based on the above aspects, this study was conducted with the aim of evaluating the effects of tDCS on the accumulation of lipids and hepatic inflammatory parameters (factors that are involved with the development of MAFLD) in an experimental model of obesity.

## Material and methods

### Animals

For the development of this study, we used biological samples from a previous research protocol, which used forty adult (60 days old) male Wistar rats, weighing 200–250 g^20^. The animals were randomized by weight and length measurements and housed in three or four per polypropylene cage with sawdust-covered floors. Rats were maintained on a standard 12-h light/dark cycle (lights on at 7:00 a.m.), in a temperature-controlled environment (22 ± 2 °C). All experiments and procedures were approved by the Institutional Ethics Committee for the Use of Animals of the institution Hospital de Clínicas de Porto Alegre (protocol #2011-0455 and #2018-0640). The procedures for scientific animal’s use were conducted in accordance with the Guide for the Care and Use of Laboratory Animals (8th ed., 2011) and law #11.794 (Brazil, 2008). The execution of this study was carried out in accordance with the ARRIVE guidelines (Animal Research: Reporting of In Vivo Experiments). The number of animals used in this experiment was the necessary number to produce reliable scientific data.

### Study design

**Figure 1:**
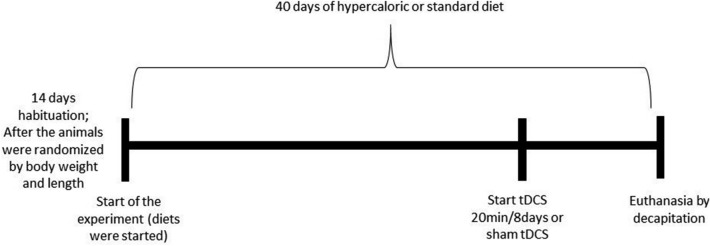
Experimental design. *tDCS* transcranial direct current stimulation.

The rats were divided into groups of three or four animals per cage and habituated to the maintenance room for two weeks before the start of the experiment, as described in a previous publication^20^. After the habituation period, the animals were randomly selected for body weight and length measurements and subsequently allocated to four treatment groups (n = 10/group): standard diet plus sham tDCS treatment (SDS), standard diet plus active tDCS treatment (SDT), hypercaloric diet plus sham tDCS treatment (HDS), or hypercaloric diet plus active tDCS treatment (HDT). After 40 days on a hypercaloric diet and/or standard diet, the experimental groups received active or sham tDCS treatment for 20 min each day for eight consecutive days. Two days later, the rats were killed by decapitation and the liver was removed and stored in an ultrafreezer at − 80 °C to carry out the proposed evaluations (Fig. 1).

As previously reported, a number of steps were taken to control for possible measurement bias^20^. The researchers were blind to the numbers assigned to the animals' test boxes, so it was impossible to distinguish between groups that received active or sham tDCS treatment. This blinding was maintained for the analysis carried out in the present study.

### Experimental diets and food intake

The animals in the SDS and SDT groups received a standard rodent chow diet (Nuvilab CR-1, Quimtia S.A., Brazil) with an energy content of 2.93 kcal/g (information provided by the manufacturer). This diet consisted of 55.0% carbohydrates, 22.0% protein, 4.5% fat and 18.5% other constituents (fiber and vitamins). The hypercaloric diet (cafeteria diet) was offered to the experimental groups HDS and HDT for inducing obesity in rodents, mimicking the genesis and metabolic responses observed in the disease in humans. The diet used in this study was adapted from the hypercaloric or western diet, previously described by Estadella et al.^23^. The foods offered in the hypercaloric diet are palatable and flavored, characteristics of a comforting food, including crackers, sausages, snack foods, wafers, condensed milk and soda. The nutritional composition of this diet was approximately 60.0% carbohydrates, 20.0% lipids, 15.0% protein and 5.0% other constituents (sodium, calcium, vitamins, preservatives and minerals), providing 4.186 kcal/g (solids) and 0.42 kcal/mL (soda drink; calculations based on information provided by the manufacturer on the package label). Animals exposed to the hypercaloric diet also had access to standard food and water. The diet offered to animals in all groups of experiments was replaced daily by fresh food. The animals had water and food supplied ad libitum during the experiment period.

Food and caloric consumption were calculated according to de Oliveira et al*.*^24^. Food was weighed daily to assess the amount of food consumed per cage. In order to evaluate the amount of food consumed in each cage the caloric intake was evaluated during the experiment and the daily dietary intake was calculated as the difference between the diet offered and the leftovers collected from the cage. Food was weighed daily to assess the amount of food consumed per cage. The weight in grams was then converted to Kcal. Using a digital scale, the daily food consumption (in grams) was calculated for each cage (amount of food placed in the feeders minus the remaining food, after which the weekly average and cumulative food consumption assessment was performed (total food consumption).

### Transcranial direct current stimulation (tDCS)

After 40 days on a standard or hypercaloric diet, the animals in the active treatment groups underwent 20 min of bicephalic tDCS, every afternoon, for 8 consecutive days, as described by Macedo et al.^14^. The electrodes were positioned, fixed to the head with surgical tape (Micropore™), and covered with a protective mesh to prevent removal. A constant direct current of 0.5 mA was delivered from a battery-powered stimulator using pediatric electrocardiogram electrodes with conductive adhesive hydrogel. The rats’ heads were shaved for firmer adherence and the electrodes were trimmed to 1.5 cm^2^ for better fit, and thus emulate the tDCS method used in human food craving studies^19^. The stimulation target was the DLPFC, because modulation of this area has been shown to reduce food craving^22,25^. The center of the anodal electrode was placed over the right DLPFC and the cathode over the left DLPFC. According to an earlier animal safety study, a current density higher than 142.9 A/m^2^ is associated with brain lesions^26^. Considering this threshold, the stimulation parameters were set, resulting in a current density of 33.4 A/m^2^. In addition, a constant current of 1 mA of intensity causes skin lesions. For sham stimulation, the electrodes were placed and fixed in the same position as for the active stimulation; however, the stimulator remained in the “off” position throughout the procedure^20^. In order to deliver the current, animals had to be immobilized with a cloth for the total duration of stimulation.

### Quantitative analysis of liver fat deposition

In order to analyze the hepatic lipid content, the previously frozen liver tissue samples were thawed on ice and homogenized in phosphate buffer (20 mg of tissue/mL). Triglyceride concentration, total cholesterol and total lipid accumulation were evaluated using the Nile Red method^27^. The hepatic triglycerides and total cholesterol were assayed enzymatically by colorimetry (Labtest Diagnóstica S.A, Brazil). All analyses were done in duplicate.

The total lipid concentration was determined using the modified protocol of Gómez-Lechón et al.^27^. In brief, liver tissue fragments, previously homogenized in phosphate buffer, were incubated with 1 μl of Nile Red solution (1 mg/mL in acetone) at 37 °C for 15 min. Fluorescence was measured at 488 nm excitation and 550 nm emission (SpectraMax M3). All analyses were done in duplicate. The obtained values were normalized to total protein present in the homogenate^28^. The results were expressed as fluorescence/μg protein.

### Protein evaluation of hepatic inflammatory mediators

Using the animals' liver tissue, we performed the evaluation of the following inflammatory markers: tumor necrosis factor (TNF) -α, interleukin (IL)-1β and IL-10 (Invitrogen, USA). These assessments were carried out using the ELISA technique. All analyses were done in duplicate and according to the manufacturer's instructions. Absorbance was measured in a spectrophotometer at 450 nm (Zenyth 200 rt). Cytokine concentrations were determined using a standard curve and the results were expressed in pg/mg protein. The data obtained were used to calculate the balance between the pro- and anti-inflammatory cytokines, called the inflammatory index.

### Statistical analysis

Normality was verified for all variables using the Shapiro–Wilk test, and parametric tests were used to analyze data with normal distribution. Inflammatory markers were analyzed by two-way ANOVA. For the analysis of hepatic triglycerides, the one-way ANOVA test followed by Bonferroni corrections was applied. A generalized estimating equation (GEE) followed by Bonferroni corrections was performed to analyze the results of cumulative food consumption. Quantitative variables were expressed as mean ± standard error of the mean (SEM). *p* < 0.05 was considered statistically significant. Data were analyzed using the Statistical Package for Social Sciences 18.0 (SPSS Inc., USA).

## Results

### Assessment of cumulative food consumption

**Figure 2:**
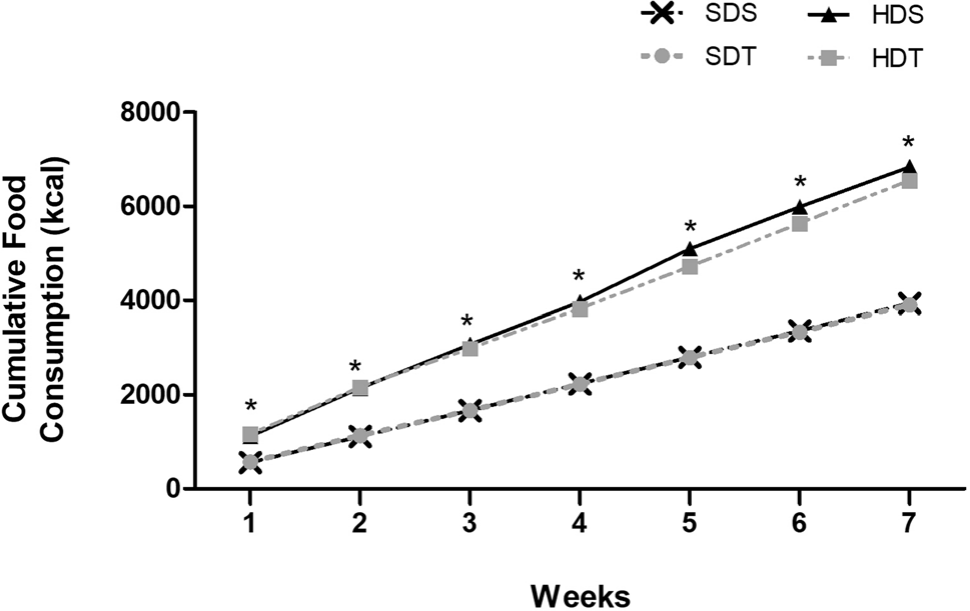
Assessment of cumulative food consumption. Data expressed as mean ± SEM, (n = 10/group). *SDS* standard diet plus sham tDCS treatment, *SDT* standard diet plus tDCS treatment, *HDS* hypercaloric diet plus sham tDCS treatment, *HDT* hypercaloric diet plus tDCS treatment. *Significant difference between SDS and SDT groups and HDS and HDT groups (GEE, *p* < 0.05).

The cumulative consumption of the diets offered to the respective experimental groups were evaluated during the experimental period. As expected, the animals that received a high-calorie diet (HDS and HDT) had a significantly higher caloric intake from the first week of the experiment in relation to the SDS and SDT groups that received a standard diet (GEE test, *p* < 0.05, Fig. 2A). No interaction effect was observed between the animals that received treatment with tDCS (SDT and HDT) and the groups that received sham tDCS treatment in the cumulative food consumption, during the experimental period (GEE test, *p* > 0.05, Fig. 2A).

### Analysis of fat deposition in liver tissue

**Figure 3:**
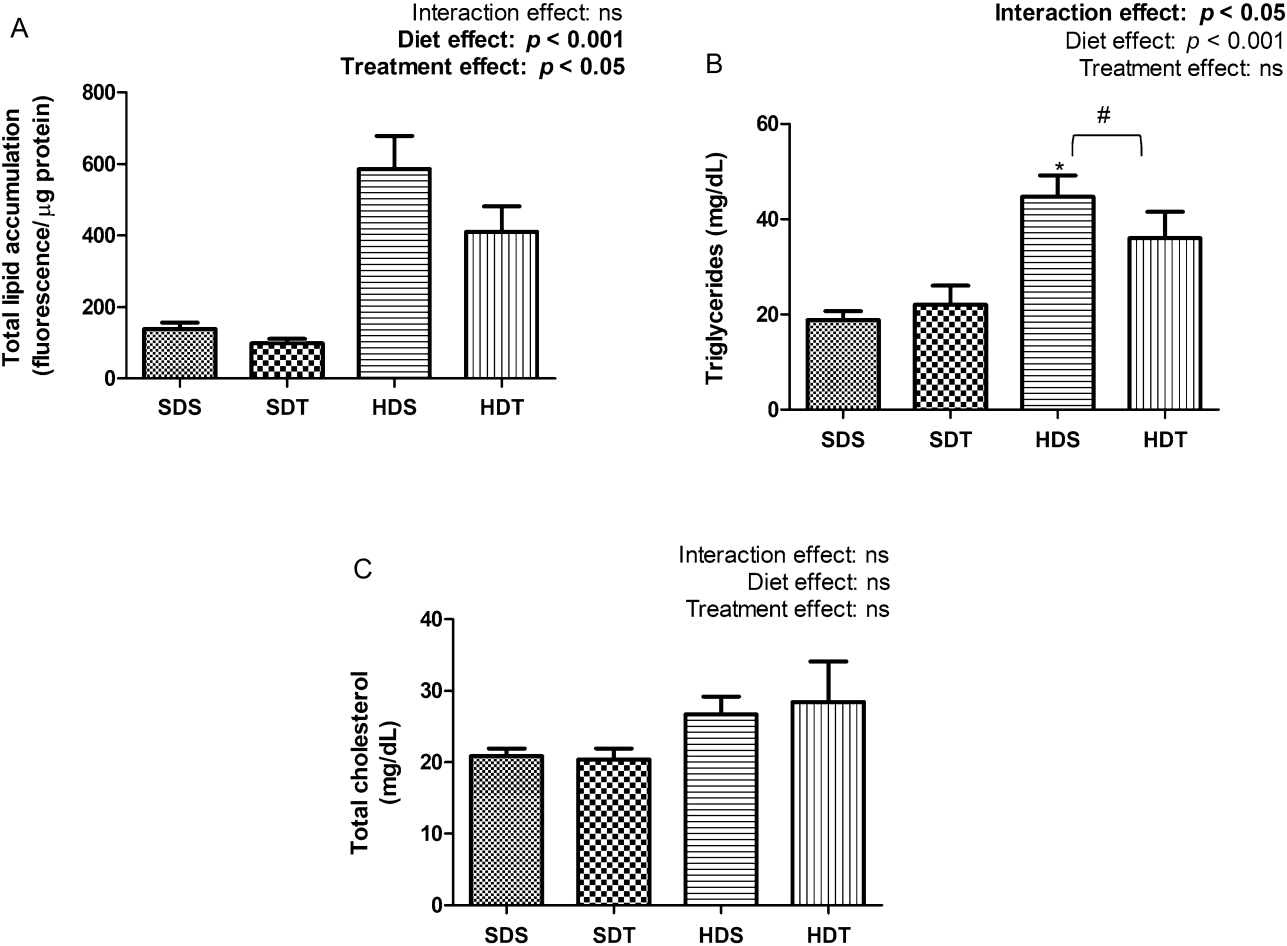
Quantitative analysis of liver fat deposition. (**A**) Total lipid accumulation (fluorescence/μg protein), (**B**) triglyceride (mg/dL) and (**C**) total cholesterol (mg/dL). Data expressed as mean ± SEM (n = 10/group). *SDS* standard diet plus sham tDCS treatment, *SDT* standard diet plus tDCS treatment, *HDS* hypercaloric diet plus sham tDCS treatment, *HDT* hypercaloric diet plus tDCS treatment. *Significant difference between HDS and SDS groups (*p* < 0.001). ^#^Significant difference between HDS and HDT groups (*p* < 0.05).

In the quantitative analysis of hepatic lipid deposits, the animals that received the hypercaloric diet (HDS and HDT) showed a significant increase compared to the experimental groups that received the standard diet (SDS and SDT). Animals that received treatment with tDCS (SDT and HDT) showed a significant reduction in hepatic lipid accumulation compared to groups that received sham tDCS treatment (two-way ANOVA diet effect, *p* < 0.001 and *p* < 0.05, respectively, Fig. 3A).

There was an interaction effect on the accumulation of triglycerides in the hepatocytes of the experimental groups under study (*p* < 0.05). We demonstrated that the HDS group showed a significant increase in hepatic triglyceride concentration compared to the SDS group (adjusted by Bonferroni, *p* < 0.001, Fig. 3B). Treatment with tDCS significantly reduced the concentration of liver triglycerides in animals in the HDT group compared to the HDS group (adjusted by Bonferroni, *p* < 0.05, Fig. 3B). There were no significant differences between the experimental groups in the hepatic concentration of total cholesterol (Fig. 3C).

### Liver inflammatory parameters

**Figure 4:**
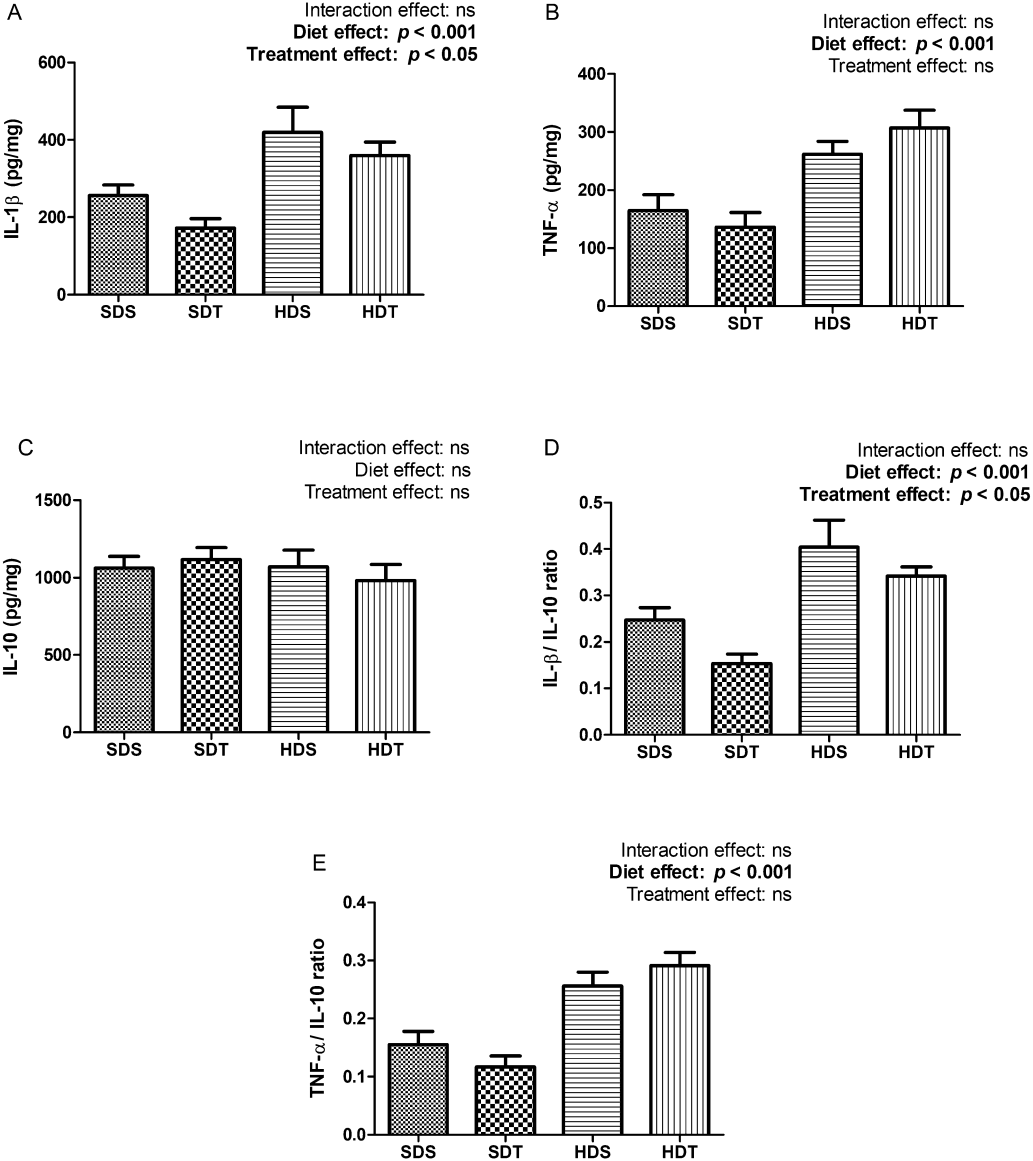
Hepatic inflammatory parameters. (**A**) IL-1β levels, (**B**) TNF-α levels, (**C**) IL-10 levels, (**D**) IL-1β/IL-10 ratio and (**E**) TNF-α/IL-10 ratio. Data expressed as mean ± SEM (pg/mg of protein), (n = 10/group). *SDS* standard diet plus sham tDCS treatment, *SDT* standard diet plus tDCS treatment, *HDS* hypercaloric diet plus sham tDCS treatment, *HDT* hypercaloric diet plus tDCS treatment.

There was a significant increase on the hepatic concentration of the pro-inflammatory markers IL-1β and TNF-α (two-way ANOVA diet effect, *p* < 0.001 for all parameters, Fig. 4A,B, respectively) in rats with access to the hypercaloric diet compared to the SD groups (SDS and SDT). Treatment with tDCS significantly reduced the concentration of IL-1β (two-way ANOVA treatment effect, *p* < 0.05, Fig. 4A) compared to the groups that received sham tDCS treatment (SDS and HDS). There were no significant changes in the hepatic concentration of IL-10 among the experimental groups under study (Fig. 4C).

In the assessment of the hepatic inflammatory index, there was a significant increase in the IL-1β/IL-10 and TNF-α/IL-10 ratios (two-way ANOVA diet effect, *p* < 0.001 for all parameters, Fig. 4D,E, respectively) in the groups that received the hypercaloric diet (HDS and HDT) compared to animals that received the standard diet (SDS and SDT). There was a significant effect of active tDCS treatment on the IL-1β/IL-10 index (two-way ANOVA treatment effect, *p* < 0.05, Fig. 4D) compared to the experimental groups that received sham tDCS treatment (SDS and HDS).

## Discussion

This study was carried out in an experimental model of obesity with the aim of evaluating the effect of the application of tDCS on the accumulation of lipids and liver inflammatory mediators, factors associated with the development of MAFLD. We demonstrated that bicephalic tDCS treatment significantly reduces the accumulation of triglycerides in animals that were fed a hypercaloric diet. The application of active tDCS was also able to significantly reduce the accumulation of lipids, IL-1β and the IL-1β/IL-10 ratio compared to animals that received sham tDCS treatment. Additionally, we report that experimental groups fed a hypercaloric diet showed a significant increase in the cumulative food consumption, deposits of liver lipids, liver triglycerides, pro-inflammatory mediators and inflammatory indexes compared to animals fed with a standard diet. Limiting the consumption of calorie-dense foods is essential for preventing the onset of many chronic diseases. Evidence suggests that the obese subject’s behavior may be related to abnormal brain function, characterized by dysfunctional inhibitory control and decision-making capacities. In this sense, the application of the non-invasive tDCS technique has been investigated in obesity.

Obesity-related steatosis, or MAFLD, represents a huge public health problem and is related to abnormal accumulation of lipids in the liver^29^. In a previous paper published with data from the same animals as from the current study, it was reported that rats fed the hypercaloric diet developed obesity, with a significant increase in the amount of visceral adipose tissue and liver weight^20^. In the present study, we observed that animals fed hypercaloric diet showed a significant increase in the cumulative food consumption, deposition of liver lipids and triglycerides compared to groups that received a standard diet. These results are in accordance with previous studies published in the literature^30–32^. Steatosis occurs whenever the import or synthesis of fat exceeds fat export or degradation^29,33^. Triglyceride (triacylglycerol) is a type of fat and its accumulation is the hallmark for the presence of fatty liver, and in this study significant changes in its concentration in animals fed a hypercaloric diet have been reported. The extent of triglyceride accumulation has been the basis for grading the severity of steatosis in MAFLD^33^. A limitation of the current study was the impossibility of performing histopathological assessment of liver damage. However, according to the literature, rats fed with high-fat content develop steatosis, characterized by a significant increase in the hepatic lipid content, in the first two weeks of the experiment, decreasing later, and reappearing between the 6th–12th weeks^32^. Non-alcoholic steatohepatitis usually develops after the 12th week of exposure to a high-fat diet^32^. It is known that high caloric intake is related to metabolic inflammation caused by several clinical conditions. Fundamental to the control of food intake is the interaction between the central nervous system and peptide hormones released within the circulation of the gastrointestinal tract^34^. Peripheral signals generated by the liver are received and integrated by neural circuits, mainly in the hypothalamus and brain stem, acting in the regulation of energy balance and metabolic homeostasis^34,35^. This occurs via hypothalamic neuropeptides, central nutrient sensing, and the action of peripheral hormones on the central or peripheral nervous systems^34^. Abnormalities in the activation of these regulatory pathways contribute to increasing hepatic secretion of very low-density lipoprotein, dyslipidemia and insulin resistance^34^.

From this information, palatable foods rich in fat/sugar can trigger an irresistible motivation for consumption known as “food craving”, and this could be a predictor of relapse or weight regain in obese patients^14,21^. The decision for food consumption is influenced by several factors, including the levels of blood sugar, hormonal changes, emotional state, and physical activity. However, the action of eating is finally processed in neural networks associated with decision-making, that involves mainly prefrontal cortical areas. The DLPFC, as one part of the prefrontal cortex, is involved in the processing of reward perception, motivation, and decision making^36^. Thus, the stimulation of this area through the application of tDCS is able to decrease food consumption and food craving, it can be suggested that stimulation of DLPFC modulates different pathways involved in food consumption^19,20,36^.

In this study, we reported that cumulative food consumption was significantly higher over the experimental period in rats fed a hypercaloric diet compared to the group of animals fed only standard chow. This study was developed from biological samples previously collected^20^. In a previous paper published with data from these same animals, was demonstrated that animals that consumed a hypercaloric diet showed an increase in the weight delta (HDS and HDT groups), Lee index (HDS group), relative liver weight (HDS and HDT groups) and visceral adipose tissue weight (group HDS), confirming the induction of obesity^20^. In conclusion, it was observed that bicephalic tDCS treatment reverses the increase in the Lee Index and the relative visceral adipose tissue weight^20^. However, no interaction effect was observed between the offered diet and the application of tDCS on cumulative food consumption. In the current study, the application of active tDCS significantly reduced the accumulation of hepatic triglycerides in animals fed with a hypercaloric diet compared to the group that received sham tDCS treatment, and these changes occurred regardless of changes in food intake. After ingestion of a meal, the presence of nutrients in the gastrointestinal tract initiates complex neural and hormonal responses through the gut–brain–liver axis^37^. The gastrointestinal tract possesses intrinsic neural plexuses that allow a significant degree of autonomy over functions including digestion, nutrient absorption and the elimination of waste, in which the central nervous system provides extrinsic neural inputs that regulate, modulate, and control these functions^38^. The activation of these mechanisms are important for the energetic and metabolic balance, among them the regulation of glucose and insulin levels and maintenance of circulating and hepatic lipid homeostasis^34^. The activation of these homeostatic mechanisms regulates glucose and insulin levels. However, this regulatory system quickly fades in the face of continued ingestion of a fat-rich diet, causing the development of several comorbidities such as obesity, MAFLD, and diabetes^37^. Numerous factors can influence the decision of food consumption; in this sense, a possible approach to regulate food craving might be to interfere in this decision-making process, changing the activity of the DLPFC through the application of tDCS^14,19^. It is important to highlight that tDCS, in addition to modifying the activity of cortical areas that are located directly under the electrodes, also modulates distant areas, possibly due to primary interconnections^14^. In this study, we demonstrated that tDCS treatment exerts its beneficial effect on the accumulation of liver fat in obese rats; based on the above, this is due to an interaction between the gut–brain–liver axis, although these mechanisms are not yet completely elucidated. There is scant research on animal models using tDCS in food craving and to our knowledge, there are no studies evaluating its effect on the liver's energy metabolism and on the modulation of the liver inflammatory response.

The high prevalence of MAFLD is strongly linked to obesity. These clinical conditions predispose a myriad of comorbidities, including type 2 diabetes mellitus, cardiovascular disease, and malignancy^39^. Moreover, in MAFLD, obesity was shown to be an independent predictor of fibrosis progression, development of nonalcoholic steatohepatitis, and mortality^39^. It is known that in steatosis there is an increase signaling of the transcription factor NF-κB (nuclear factor-kappa B), that induces the production of pro-inflammatory mediators^8^. These contribute to the recruitment and activation of Kupffer cells (resident hepatic macrophages), responsible for mediating the inflammatory process triggered by this clinical condition^8^. In this study we demonstrated that experimental groups fed a hypercaloric diet showed a significant increase in the hepatic concentration of IL-1β and TNF-α and IL-1β/IL-10 and TNF-α/IL-10 ratios compared to animals that received a standard diet. These data are in agreement with previous reports in the literature, which demonstrate that in obesity and/or MAFLD, a low-grade inflammatory state occurs with increased body fat, particularly visceral fat, impairing metabolic control and normal body weight^40,41^. We also report that treatment with tDCS significantly reduces the hepatic concentration of IL-1β and IL-1β/IL-10 index compared to the experimental groups that received sham tDCS treatment. As far as we know, there are no studies evaluating the effects of applying tDCS on hepatic inflammatory parameters in an experimental model of obesity or MAFLD. As a result of the beneficial effect of the application of tDCS on the accumulation of hepatic lipids, we report a downregulation of hepatic inflammatory markers. Moreover, the results previously published from these same animals demonstrated that the application of tDCS decreases the levels of TNF-α and IL-1β in the cerebral cortex of animals fed with a hypercaloric diet compared to groups that received a standard diet, suggesting that the bicephalic tDCS treatment could modulate the inflammatory parameters in an experimental model of obesity^20^.

To our knowledge, this is the first study that evaluates the effects of application of tDCS on the accumulation of lipids in the liver and hepatic inflammatory parameters in an experimental model of obesity. We thereby demonstrate that animals fed with a hypercaloric diet and treated with bicephalic tDCS over the DLPFC, significantly reduce the accumulation of hepatic triglycerides, the most evident type of fat in fatty livers. Additionally, we report that application of active tDCS was also able to significantly reduce the accumulation of total lipids, IL-1β and IL-1β/IL-10 index compared to animals that received sham tDCS treatment. Yet, the mechanisms by which tDCS exerts its effects are not fully understood, indicating that further studies are needed to guide the understanding of these findings. This study has a translational potential in which the application of tDCS over the DLPFC in obese patients with MAFLD can decrease food consumption and food craving, preventing progression of liver damage.

## Abbreviations

DLPFC: Dorsolateral prefrontal cortex
HDS: Hypercaloric diet plus sham tDCS treatment
HDT: Hypercaloric diet plus tDCS treatment
IL: interleukin
MAFLD: Metabolic associated fatty liver disease
NAFLD: Non-alcoholic fatty liver disease
NF-κB: nuclear factor – kappa B
FDA: Food and Drug Administration
SDS: Standard diet plus sham tDCS treatment
SDT: Standard diet plus tDCS treatment
TNF-α: tumor necrosis factor -α
tDCS: Transcranial direct current stimulation
